# Prospective study of high-risk, *BRCA1/2*-mutation negative women: the *‘negative study’*

**DOI:** 10.1186/1471-2407-14-221

**Published:** 2014-03-25

**Authors:** Joanne Kotsopoulos, Kelly Metcalfe, Jill Alston, Dina Nikitina, Ophira Ginsburg, Andrea Eisen, Rochelle Demsky, Mohammad Akbari, Kevin Zbuk, Steven A Narod

**Affiliations:** 1 Familial Breast Cancer Unit, Women’s College Research Institute, 790 Bay St, 7th Floor, Toronto, ON M5G 1 N8, Canada; 2 Dalla Lana School of Public Health, University of Toronto, 155 College St, Toronto, ON M5T 3 M7, Canada; 3 Lawrence S. Bloomberg Faculty of Nursing, 155 College St, Toronto, ON M5T 3 M7, Canada; 4 Toronto-Sunnybrook Regional Cancer Center, 2075 Bayview Ave, Toronto, ON M4N 3 M5, Canada; 5 Department of Obstetrics and Gynecology, Division of Gynecologic Oncology, University of Toronto, 92 College Street, Toronto, ON M5G 1 L4, Canada; 6 Population Health Research Institute, Hamilton Health Sciences, McMaster University, 237 Barton Street East, Hamilton, ON L8L 2X2, Canada

**Keywords:** Family history, Breast cancer, Risk factors, Prevention, *BRCA1/2* mutation, Diet, Lifestyle, Hormones, Prospective cohort, Screening

## Abstract

**Background:**

We previously reported that women from high-risk families who tested negative for a *BRCA1* or *BRCA2* (*BRCA1/2*) mutation were four times more likely to develop breast cancer compared to women in the general population. Preventive measures and risk factors for breast cancer development in these high-risk women have not been evaluated to the same extent as *BRCA1/2* positive women. Further, there is virtually no scientific evidence about best practices in their management and care. The proposed study will examine a role of genetic and non-genetic factors and develop the systems and parameters for the monitoring and surveillance necessary to help establish guidelines for the care of this high-risk population.

**Methods/Design:**

To achieve our goals, we will assemble and follow a Canadian cohort of 1,000 cancer-free women with a strong family history breast cancer (defined as two or more relatives affected by breast cancer under the age of 50, or three or more relatives diagnosed with breast cancer at any age from one side of the family and with no *BRCA1/2* mutation in the family). All eligible participants will be mailed a study package including invitation to participate, consent form, a research questionnaire to collect data regarding family history, reproductive and lifestyle factors, as well as screening and surgery. Usual dietary intake will be assessed by a diet history questionnaire. Biological samples including toenail clippings, urine and blood samples will be collected. These women will be followed every two years by questionnaire to update exposure information, screening practices, surgical and chemoprevention, and disease development.

**Discussion:**

Findings from this study will serve to help establish clinical guidelines for the implementation of prevention, counseling, and treatment practices for women who face an elevated risk of breast cancer due to family history, but who do not carry a *BRCA1/2* mutation.

## Background

Approximately 10-15% of all breast cancers are estimated to have a hereditary component [1,2] and a family history of breast cancer is associated with an increased risk of the disease [3]. Risk increases with the number of first-degree relatives affected and with decreasing age at onset of the affected family members [4]. In Canada and other countries, women with significant family history of breast and/or ovarian cancer are eligible for genetic testing for mutations in either one of the two breast cancer susceptibility genes, *BRCA1* and *BRCA2* (*BRCA1/2*). Genetic screening provides the opportunity for women identified as carriers of a deleterious mutation to consider various risk-reducing options (e.g., prophylactic mastectomy and/or oophorectomy, tamoxifen) [5] or intensive screening (e.g., annual MRI, mammography) [6]. Despite this, the majority of women with a family history of breast cancer who undergo genetic testing do not carry a *BRCA1/2* mutation [7]. This finding is often interpreted as ‘non-informative’ because the residual cancer risk is high which represents a difficult challenge in the clinic given that there is no consensus on whether high-risk women who are *BRCA* mutation-negative should follow the same preventive measures as women with an identified *BRCA1/2* mutation.

We previously evaluated the risk of breast cancer among 1,492 women with a strong family history of breast cancer (i.e., two or more breast cancers under the age of 50, or three or more breast cancers at any age), but who tested negative for a *BRCA1/2* mutation [8]. We reported a four-fold increased risk of breast cancer among these women, equivalent to a lifetime risk of 40%. Age-specific standardized-incidence ratios (SIRs) ranged from 14.9 in females aged 25-39 to 3.0 in females aged 60 and over suggesting greater risks for younger women. There was a significant excess in the number of observed breast cancers for all subgroups of cases stratified by age (65 breast cancers observed, 15 expected). Unlike *BRCA1/2* mutation carriers, no elevated risk of ovarian cancer or any other cancer was found in these females.

### Rationale of the *‘Negative Study’*

There are currently no protocols in place for the counseling of women from high-risk families without *BRCA1/2* mutations. Preventive measures and risk factors for breast cancer development have not been evaluated to the same extent and there is currently virtually no scientific evidence about best practices in their management and care. Although risks in these women are not as high as those for *BRCA* mutation carriers (40% lifetime risk compared to 80%), they remain significantly higher than risks in the general population (11% lifetime risk) and thus highlight the need to improve surveillance and preventive measures specifically for these women [9-11]. The prevention of breast cancer in these high-risk women has a special dimension. First, they are not generally considered to be at sufficiently high-risk that preventive mastectomy is warranted. Second, the risk for breast cancer is site-specific, that is, they are not at increased risk for ovarian cancer [8,12]. Until now, the principal risk-reducing option in high-risk women has been the use of tamoxifen or raloxifene although recent findings suggest superior effectiveness of exemestane and anastrozole in reducing the incidence of breast cancer in high-risk postmenopausal women [13-15]. Yearly imaging with MRI has been shown to be a more sensitive means of screening for breast cancer than annual mammography for women at a high risk of cancer due to a *BRCA1* or *BRCA2* mutation, or at moderate risk of cancer due to a strong family history [16-19]. Despite this, our group recently found little evidence to support the recommendation that annual MRI screening adds benefit beyond annual mammography and ultrasound for the low/moderate risk population (unpublished data). It is clear that guidelines around prophylaxis and the appropriate use of surveillance and prevention methods are needed. Further, a role of modifiable factors is unknown.

To this end, we initiated a multi-centered, hospital-based, cohort study of high-risk/mutation negative women identified through genetic counseling clinics in Ontario *(‘Negative Study’*) (funded by the Canadian Breast Cancer Foundation). This study was designed to address various research questions including: 1) what factors (including hormonal, lifestyle factors, surgery, chemoprevention, biological markers, and mutations in other predisposing genes) influence risk for breast cancer development; 2) to what extent are screening or preventive measures being utilized in this high-risk group; 3) what are the benefits of preventive programs in reducing the incidence of breast cancer development in this high-risk group?

Approximately 20-25% of familial breast cancers can be attributed to a germline mutation in *BRCA1* or *BRCA2*[7,20]. Thus, after accounting for the known high-risk breast cancer loci, more than 75% of the familial risk of disease still remains unexplained [21] resulting in an under-representation of the number of women at-risk, and subsequently, an increase in the number of women developing and possibly dying of cancer. Given this, there is a need to integrate testing for germline mutations in other known breast and ovarian cancer predisposing genes that may collectively account for an additional 30% of the hereditary breast cancers. Based on technological advancements with DNA sequencing technology, along with the significant drop in the cost of sequencing, it is time to consider a comprehensive gene panel for hereditary breast and ovarian cancer genetic testing. This model should include testing for mutations in the 25 known Breast and Ovarian Cancer Predisposing (i.e., BrOCaP) genes that are involved in the balance between cell growth and cell death, as well as maintaining genome integrity [22-36]. A complete list of all the BrOCaP genes and their chromosomal location is presented in Table 1. Given their important role in breast cancer predisposition, it is timely that genetic testing include the evaluation of mutations in these additional 23 genes, and not only mutations in *BRCA1* and *BRCA2*, which represents the current testing protocol in cancer genetic clinics.

**Table 1:** List of genes included in the Breast and Ovarian Cancer Predisposing (BrOCaP) gene panel

**Gene**	**Chromosome**	**Gene**	**Chromosome**	**Gene**	**Chromosome**
***ATM***	chr11	***MLH1***	chr3	***PTEN***	chr10
***BARD1***	chr2	***MRE11A***	chr11	***RAD50***	chr5
***BRCA1***	chr17	***MSH2***	chr2	***RAD51C***	chr17
***BRCA2***	chr13	***MSH6***	chr2	***RAD51D***	chr17
***BRIP1***	chr17	***MUTYH***	chr1	***STK11***	chr19
***CDH1***	chr16	***NBN***	chr8	***TP53***	chr17
***CHEK2***	chr22	***PALB2***	chr16	***XRCC2***	chr7
***EPCAM***	chr2	***PPM1D***	chr17		
***FAM175A***	chr4	***PMS2***	chr7		

Our group has developed and previously validated the BrOCaP gene panel sequencing in our molecular genetics research laboratory at the Women’s College Research Institute for the screening of all the known BrOCaP genes. This panel targets 411 coding exons (plus 10 bp from the introns in each side of an exon) of 25 genes (Table 1) in 914 amplicons. These constitute about 80 Kbp of the human genome. We have validated the reliability of our BrOCaP gene panel sequencing by re-identifying 91 of the 92 previously known mutation carriers in 16 different genes in this panel. Each of the investigated carriers in this validation study had a different mutation including single nucleotide changes, insertion and deletions with sizes range from 1 to 55 bps. The only mutation that could not be re-identified was a 55 bp deletion (*BRCA1* c.5279_5333del55) which is difficult to detect with short read sequences (unpublished data). The prevalence and importance of genetic variation in these additional genes has yet to be evaluated in a large sample of women from families with a strong family history of breast cancer. Using this panel, we plan to evaluate the prevalence of these mutations within our cohort.

Based on knowledge of the etiology of both hereditary and sporadic breast cancer, we have *a priori* selected several risk factors and biological markers which we plan to evaluate in our study (reviewed in [37-39]). These have been summarized in Table 2. This is not meant to be a comprehensive list but instead an overview of potentially important variables. Briefly, we will evaluate the role of reproductive factors including age at menarche, parity, breastfeeding, age at first full-term pregnancy and age at menopause, as well as a role of exogenous hormone use including oral contraceptives (i.e., OC) and hormone replacement therapy (i.e., HRT). The ability to modify risk in this group of high-risk women without a *BRCA1/2* mutation through diet and lifestyle represents a critical area of research that warrants evaluation and has important implications for prevention. Given the lack of evidence for the aforementioned risk factors in this high-risk population, this represents a novel opportunity to establish a preventive role of modifiable risk factors. Thus, we will evaluate the relationship with lifestyle factors including body and physical activity [40,41] as well as dietary exposures such as alcohol consumption [42] and folate status [43].

**Table 2:** Summary of variables to be assessed, examples and associated timeline

**Variable**	**Example**	**Baseline**	**Biennial follow-up**	**As needed/patient reported**
**Information collected by research questionnaire**				
Reproductive factors	Age at menarche, parity, breastfeeding	X	X	
Exogenous hormonal exposures	OC use, HRT use	X	X	
Body size – current, at age 18, 30 and 40	Weight, height	X	X	
Lifestyle factors	Physical activity, smoking status	X	X	
Screening	MRI, mammogram, breast biopsy	X	X	
Surgery	Mastectomy, oophorectomy, other	X	X	
Chemoprevention	Tamoxifen, raloxifene, etc.	X	X	
Cancer diagnosis	Breast cancer, other cancers	X	X	X
Family history	Any new cancer diagnoses	X	X	
Updated contact information	Participant, alternate contact	X	X	X
**Information collected by DHQ**				
Queries frequency of intake over the past year for 124 individual food items and provides accurate estimates of usual daily dietary intake.	Linked to the Canadian Nutrient file, this database contains average values for the content of 112 nutrients in 4,943 foods available in Canada	X	X	
**Information collected from medical records**				
Mammographic density (% density)	Quantify density using *Cumulus 4* software			X
MRI				X
Pathology for breast cancer diagnoses	Histology, stage, grade, hormone-receptor status, treatment regimens			X
**Information collected from biological samples**				
DNA	Sequencing for mutations in 23 genes in BrOCaP panel	X		X
Plasma	Sex hormone levels, nutrients	X		X
Toenail clippings	Selenium levels	X		X

Biological markers (i.e., biomarkers) of risk including mammographic density and levels of circulating sex hormone levels will also be assessed as these have been shown to be important predictors of breast cancer risk in the general population as well as other high-risk populations [44-46]. It is of interest to investigate whether various lifestyle factors may influence these biomarkers and possibly breast cancer risk. We will also evaluate the screening practices of this high-risk group of women (e.g., mammograms, MRI, ultrasound) as well as the use and effectiveness of chemoprevention agents including selective estrogen receptor modulators (SERMs) (e.g., tamoxifen) or aromatase inhibitors (e.g., exemestane).

## Methods/Design

### Study design

**Figure 1:**
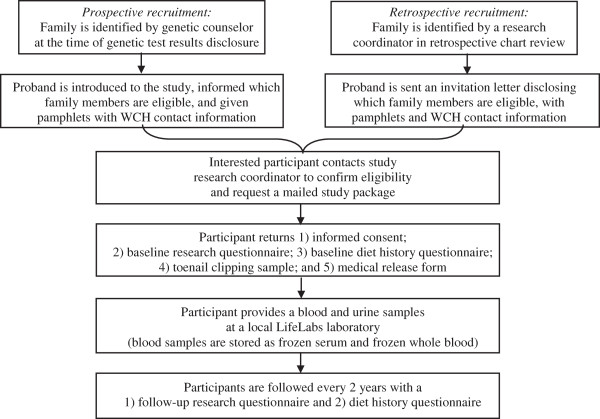
**Study design of the *‘Negative Study’.***

The *‘Negative Study’* is a large prospective cohort study on high risk women from *BRCA1/2* mutation negative families. A detailed study design is presented in Figure 1. This research is study has approval from the Research Ethics Board of each participating centre and is in compliance with the Helsinki Declaration. REB approval for this study is ongoing and was granted by Women’s College Hospital (#2010-0027-E), Princess Margaret Hospital (#09-0832-CE), Hamilton Health Sciences Centre (#11-632), Lakeridge Health Centre (#2011-033), and Sunnybrook Health Sciences Centre (#252-2012).

### Study objectives

1. To determine whether the probands of the participants in the ‘Negative Study’ carry a mutation in any of the genes in the BrOCaP gene panel.

2. To evaluate the association between hormonal and reproductive factors and the risk of breast cancer.

3. To evaluate the association between dietary and lifestyle factors and the risk of breast cancer.

4. To evaluate the level of breast cancer risk reduction with prophylactic surgery and use of selective estrogen receptor modulators or other chemopreventive agents (e.g., aromatase inhibitors).

5. To evaluate the extent to which screening measures (e.g., MRI, mammography, ultrasound) are being utilized and whether they influence breast cancer risk.

6. To evaluate the association between various biomarkers of risk (i.e., circulating sex hormone levels, mammographic density) and the risk of breast cancer.

### Study population and eligibility

#### Recruitment of eligible participants

**Figure 2:**
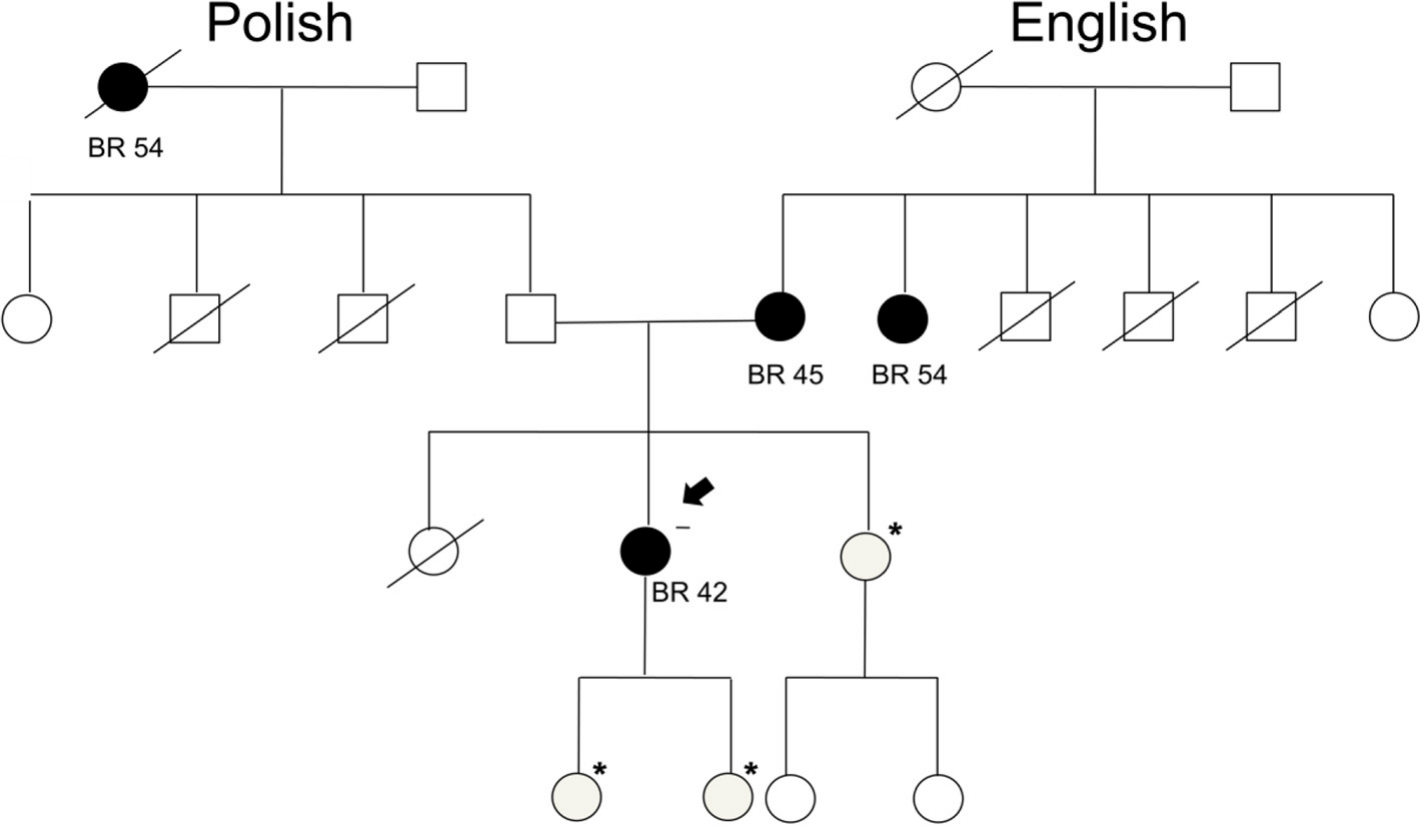
**Pedigree/identifying eligible participants.** Figure 2 displays a pedigree in which the woman with the arrow is the proband and the women marked with * are potentially eligible for the study based on the eligibility criteria. The selected individuals all 1) have a first degree relative with breast cancer, 2) have two relatives diagnosed with breast cancer before the age of 50, or three at any age from one side of the family (this pedigree meets both of these criteria on the maternal side) and 3) have a relative with breast cancer that has tested negative for a *BRCA1/2* mutation (as marked by the negative sign).

Participants are currently being recruited from the following cancer genetic centres in Southern Ontario: Hamilton Regional Cancer Centre, Mt. Sinai Hospital, Sunnybrook Health Sciences Centre, Princess Margaret Hospital, Lakeridge Health and Women’s College Hospital (WCH), the latter of which is the main research site for this study. Recruitment at the cancer genetic centres occurs by one of two methods: 1) prospective enrolment in clinic or by 2) retrospective chart review (see Figure 1 for Study Design). Both methods only involve direct contact with the proband, defined as the first woman to make contact with the participating centre, from an eligible family. Potentially eligible families are those with a family history of breast cancer defined as three or more breast cancers at any age, or two breast cancers diagnosed before the age of 50 and who do not carry a *BRCA1/2* mutation. An example of an eligible family and relatives is displayed in Figure 2. Only a history of invasive breast cancer is being considered in the current study. Families are considered negative for a *BRCA1/2* mutation if one female relative with breast cancer has been confirmed by direct DNA sequencing to not be a carrier of a mutation. Families are ineligible for the study if any relative has tested positive for *BRCA1/2* mutations. Women in families that meet the above criteria are evaluated for their eligibility based on the eligibility and exclusion criteria outlined.

Eligibility criteria:

1. Between 25 – 65 years of age

2. Residing in Ontario, Canada

3. Have no personal history of any cancer

4. Have two or more female relatives with breast cancer diagnosed before the age of 50 OR three breast cancers diagnosed at any age from ONE side of the family (maternal or paternal, not both)

5. Have at least one first degree relative with breast cancer (i.e., mother, sister, daughter)

6. Have at least one relative with breast cancer receive a negative result for a BRCA1/2 mutation

7. No prophylactic mastectomy at recruitment

Exclusion criteria:

1. Women from families where a BRCA1/2 mutation has been identified after enrolment

2. Women who cannot provide a blood and urine sample at a local laboratory

The recruitment methods are:

*1. Prospective Recruitment:* Eligibility of study participation is determined at the time of genetic test results disclosure. If the proband or one of her first degree female relatives is eligible, a brief explanation of the study goals, rationale and what study participation entails is provided by the genetic counsellor. Probands that are interested in the study are given a study package with contact information for the research coordinator at WCH for additional information about the study, and are asked to contact WCH directly.

*2. Retrospective Recruitment:* The study coordinator from WCH reviews pedigrees of probands that have had genetic testing at one of the participating centres in the past and have tested negative for a *BRCA1/2* mutation. Once eligibility of the proband or her family has been determined, the proband is sent a study invitation letter describing the goals of the study and whether she is or any of her relatives are eligible. If the proband or a relative is interested in participating, she is asked to contact WCH directly using the information provided in the letter. All contacted probands who have not responded to the letter after 6 months are contacted by a research coordinator by telephone as a follow-up.

Once an eligible participant has been enrolled in the study, they are required to provide informed consent, complete a baseline research and diet history questionnaires (DHQ), provide a blood, urine and toenail clippings samples, and consent to a follow-up questionnaire every two years to update exposure information and ascertain incident diseases (Figure 1).

### Data collection

i) Exposure Data Collected By Questionnaire: Data collection by questionnaire occurs at enrolment and every 2 years thereafter until death or completion of a 10 year period, whichever comes first. All study participants are mailed a consent form, a baseline questionnaire and a DHQ at the time of enrolment. The baseline questionnaire collects extensive information on many potentially important exposures and covariates, as well as family history of cancer screening and prophylactic surgeries, and has been used by the “Risk factor analysis for Hereditary Breast Cancer” study which currently has over 15,000 women enrolled. Participants are also sent a DHQ which is a food frequency questionnaire that was developed by staff at the Risk Factor Monitoring and Methods Branch at the National Cancer Institute and reflects Canadian food availability and food fortification practices [47]. The cohort is followed biennially by questionnaire to update exposure information and ascertain disease. Women have the option to complete the follow-up by mail, email, or by a telephone interview with a trained research assistant, depending on their preference. Regular contact also ensures that there is the most up to date contact information for the participants and an alternate contact whom we may contact in case of loss to follow-up or death. A summary of variables, examples of each, and the associated timeline for collection is displayed in Table 2.

ii) Biological Specimen Collection: Participants have the option to have their samples collected at one of several LifeLabs locations across Ontario. All women are encouraged to come to the centre as close to the time of completion of the baseline questionnaire as possible (within the first 2 years that they are actively enrolled in the study). Briefly, two blood samples are collected in tubes (one tube without additives and one EDTA-containing tube) labelled with the subject’s study number and delivered immediately to the laboratory for processing. Serum is collected, aliquotted and stored at -80°C. The EDTA-containing tube is inverted several times and stored at -80°C for future DNA extraction. An 80 mL urine sample is also collected, aliquotted and stored at -80°C. Samples are similarly collected at LifeLabs and are delivered to WCH every three months on dry ice. All samples are stored in -80°C freezers located in WCH which are alarmed and continuously monitored. Toenail clippings are collected at the time the baseline questionnaire is completed and are mailed to WCH in a sealed envelope along with the completed consent and questionnaire at the time of enrolment.

iii) Diagnostic Information: All study participants are asked for written permission at the time of enrolment to review their medical records and pathology reports to collect detailed information on screening, treatment, and diagnoses. Once a participant reports a cancer diagnosis during any follow-up cycle, the hospital/clinics are sent a requisition for the patient’s medical records. Necessary data will be extracted from the records by the investigator or trained study personnel on as needed basis. Cancers can be diagnosed through clinical means, including physical examination, through screening (mammography, MRI, ultrasound, CA-125) or through prophylactic surgery. To identify cases in non-respondents who died, we will request permission from the next of kin to seek medical records for incident cancers.

iv) Mammographic Density: Digital mammograms from the providing centres will be requested and computer-assisted measurement of mammographic density will be carried out using *Cumulus 4* software as previously described [48]. Measurements of the areas of dense tissue and total area are generated and percent density will be calculated.

### Laboratory analyses

i) DNA Extraction: In the WCH lab, the extracted PBL is thawed and then processed to extract archive quality germline DNA. Gentra Puregene DNA extraction kits from Qiagen are used for DNA extraction. The concentration of the extracted DNA is measured by a Nanodrop instrument. Extracted DNA will be stored in a lab standard 4°C refrigerator which allows for storage of whole DNA samples in a single tube without the need to frequently freeze and thaw the sample which will reduce its quality dramatically. This germline DNA sample will be used for all genetic tests in this cohort study.

ii) BrOCaP Gene Panel Sequencing: The BrOCaP gene panel will be tested on identified probands with breast cancer from eligible families which will allow identification of a larger proportion of mutation carriers of BrOCaP genes. DNA has been collected from these women previously at the time of their genetic testing, and is available for research purposes. All probands have provided written consent for research testing of their sample. If a proband is found to have one of the BrOCaP gene mutations, the unaffected relative who is a part of the *‘Negative Study’* cohort will also be tested for the same mutation. The BrOCaP gene panel sequencing has been developed and validated by Dr. Akbari in our molecular genetics research laboratory at WCRI for screening all the known BrOCaP genes (Table 1).

iii) Sex Hormone Levels: Plasma estrogen, androgen and progesterone levels will be assayed in the laboratory of Dr. R. Casper (Centre for Advanced Reproductive Technology, Toronto, ON, Canada) using radioimmunoassay kits (Vitros immunodiagnostics).

### Data management

The collection, storage, updating, and retrieval of all clinical data for the study subjects occur at the WCRI in Toronto. Upon consent to participate, subjects are assigned a study number which will identify their data. Names are removed from the data, and are verified and edited before entered into a computer file. The data are entered into an Access Database and are secured on a server that is only accessible to members of the centre. Appropriate backup is regularly maintained. Cyrillic 3 software (Oxfordshire, UK) is used to update pedigrees based on the information provided at follow-up. To protect the privacy of our participants, any files, biological specimens, results or questionnaires with identifying data are stored in locked cabinets for up to 15 years, after which, they will be destroyed and disposed of properly. Names are removed from biological specimens and are identified by subject number and appropriately disposed of after analysis.

### Sample size

Our target enrollment is 1,000 women. Based on preliminary data, we expect the incidence of breast cancer to be 1% per year, or 10% over a 10-year period with an expected number of 250 cancers. For all power calculations, we performed 2-sided tests with α = 0.05 and estimated the minimum detectable risk estimate at a power of 80%. Given that exposure data for the cohort to be evaluated in the current study is not yet available, we utilized exposure information from our prior publications of *BRCA* mutation carriers. For continuous variables, we calculated the RR for extreme quantiles using the formula by Chapman and Nam [49]. For the main effects of parity and weight change between ages 18 and 40, we have 80% power to detect a RR of 0.55 across extreme quintiles. For exposure variables that could not be classified into quantiles (e.g., mastectomy), the exposure was expressed as ever vs. never; we then calculated the minimum detectable RR between the most extreme categories [50]. For the main effects of OC, HRT, and tamoxifen, we have 80% power to detect a RR of 1.52, 1.54, and 0.58 comparing ever vs. never. Similarly, the RR for coffee consumption and a mastectomy were 0.47 and 0.64, respectively.

### Planned statistical analyses

Data analyses will be performed to complement specific objectives 1 through 6 outlined above. Various exposures which are planned to be assessed have been summarized in Table 2. All analyses will be carried out using SAS Version 9.1 (SAS Institute, Cary, NC, USA). For the prospective cohort analyses, we will use Cox proportional hazards models for failure-time data, using SAS PROC PHREG, to estimate relative risks and 95% CIs associated with the various exposures, as this allows for multivariate adjustment and evaluation of interactions [51]. For time-varying covariates, a new data record will be created for every questionnaire cycle at which a woman is at risk, with covariate values set at the time the questionnaire was returned. To control for confounding by age and calendar time, we will stratify jointly by age at start of follow-up and calendar year of the current questionnaire cycle. We will follow women without cancer from the date of study entry (date the baseline questionnaire is completed) until either: 1) date of last completed follow-up questionnaire; 2) a diagnosis of any cancer; 3) death from another cause; 4) loss to follow-up; or 5) prophylactic mastectomy. To avoid assumptions about linearity of exposure-response relationships, we will evaluate categorical versions (e.g., quantiles) of continuous variables. This reduces the chance that a small number of extreme observations will have an undue influence on the results. We will test for trends using quantile medians or continuous variables as appropriate. To test for pairwise interactions we will calculate interaction variables by multiplying the two risk factors or otherwise creating appropriate categorical interaction terms and entering them into the model. Likelihood ratio tests will be used to calculate *P*-values for interaction.

Multivariate modeling will take into account both plausibility of biological effects and statistical evidence of confounding. We will include well-established predictors of breast cancer in multivariate modeling (unless they are in the causal pathway). Other covariates whose biological effects or potential to confound are less certain will be examined individually in stratified analyses as applicable. We will use a 10% change in the parameter of interest as the criterion to select covariates into the final model [52].

## Discussion

Based on our earlier findings of a significantly increased risk of breast cancer among women from high-risk/*BRCA* negative families, we initiated a prospective cohort study to evaluate genetic and non-genetic determinants of risk and to determine the appropriate screening and prevention practices for these women [8]. While there are evidence-based practices for managing women with a *BRCA1*/*2* mutation, there is little evidence to guide practitioners on how women from families with breast cancer history and without *BRCA1/2* mutations should be managed [39]. Such options may include enhanced screening practices along with risk reduction options such as prophylactic surgery, as well as a role for modifiable risk factors, and chemoprevention [39]. Of critical importance is the need to determine whether other genetic factors are involved.

In the current study, priority will be given to the integration of the comprehensive BrOCaP gene panel into our protocol. Since recruitment is currently underway, we are proposing to sequence the probands of the participants for possible mutations in the additional 23 genes known to contribute to risk of breast and ovarian cancer as a first step. Should mutations be identified, this will allow us to offer relatives from these high-risk families additional genetic testing, and help identify those women who may also be at risk of developing breast cancer due to a mutation. This will be a significant finding for these families. Meanwhile, by collecting information on reproductive, hormonal and lifestyle factors, as well as chemoprevention with aromatase inhibitors or selective estrogen receptor modulators, we will be in a position to evaluate a role of modifiable factors that may lead to accurate and effective preventive interventions specifically for this high-risk group of women. We also propose to address the relationship between various purported biomarkers of breast cancer (e.g., mammographic density, sex hormone levels) and whether endogenous or exogenous exposures modify any possible relationships.

The proposed project will improve the understanding of the biology of this disease and the mechanisms through which risk factors may alter risk. More importantly, our findings stand to make significant contributions to the field of cancer prevention in high-risk/*BRCA*-negative women thus having high potential to influencing public health recommendations surrounding genetic testing, screening and prevention. The multidisciplinary approach of this study will allow for communication with other researchers and departments to share ideas and foster collaborations. In addition, the interaction with many health centers will support the translation of our research findings into clinical practice.

To date, we have six centres that are actively involved in subject recruitment and 119 women already enrolled in the study. A major limitation to recruitment has been the large number of ineligible pedigrees (mostly due to lack of *BRCA1/2* mutation status) or the lack of eligible females in the family (i.e., no eligible sisters or daughters). Additional barriers to recruitment have included the inability to contact eligible subjects directly due to the ethical need to recruit only through the proband directly, along with incorrect or missing contact information for the proband. The need for research ethics board approval at each centre has also been a constraint. Nonetheless, by including additional centres and introducing prospective recruitment, we have been able to enhance our participation dramatically. Strengths of the current study include a strict definition of ‘high-risk’ and the exclusion of families with a known *BRCA* mutation.

In summary, the establishment of this cohort study, along with the wealth of epidemiological and biological evidence to be collected, will lead to a much-needed understanding of familial breast cancer and provide evidence to guide health professionals in the management and care of women from high-risk/*BRCA*-mutation negative families. Ultimately, our goal is to develop viable strategies for risk reduction. Even though genetic factors are clearly important given these women’s familial connections to known breast cancer patients, the potential to modify cancer risk in these high-risk women will also come from enhanced knowledge of non-genetic, modifiable factors, including a role of dietary and lifestyle factors, as well as chemoprevention and screening. This information will aid in identifying preventive measures that are successful in reducing the risk of breast cancer development and will lead to future clinical guidelines for the management of these high-risk women.

## Abbreviations

BRCA1: Breast cancer susceptibility gene 1; BRCA2: Breast cancer susceptibility gene 2; DHQ: Diet history questionnaire; BMI: Body mass index; WCH: Women’s College Hospital; WCRI: Women’s College Research Institute; OC: Oral contraceptive; HRT: Hormone replacement therapy; BrOCaP: Breast and ovarian cancer predisposing.

## Competing interests

The authors declare that they have no competing interests.

## Authors’ contributions

JK is the principal investigator of the study. SAN, KM and JK contributed to the design of the protocol. JA and DN are responsible for data collection, data entry and management of the study. JK, JA and DN drafted this manuscript. All authors approved the final version of the study. OG, AE, RD and KZ participated in subject recruitment.

## Pre-publication history

The pre-publication history for this paper can be accessed here:

http://www.biomedcentral.com/1471-2407/14/221/prepub
